# Medical Entomology for Students, 5th Edition

**DOI:** 10.3201/eid2008.131738

**Published:** 2014-08

**Authors:** Michael L. Levin

**Affiliations:** Centers for Disease Control and Prevention, Atlanta, Georgia, USA.

**Keywords:** medical entomology, textbook, vector-borne infections, students, parasites, arthropods, Diptera

In the ever-changing field of medical entomology, Mike Service’s textbook, Medical Entomology for Students, is now in its fifth edition. The usefulness, adaptability, popularity—and, therefore, longevity—of this textbook is remarkable among the large variety of books on the subject published during the past 20 years.

As in earlier editions, the book is concisely written to provide basic information about recognition, biology, and control methods of arthropods that affect human health—parasites, nuisance pests, and vectors of human diseases—without delving into too much detail and nuance. Almost two thirds of the volume is dedicated to medically important members of the order Diptera (true flies, mosquitoes, and sandflies), and separate sections are devoted to the biology, morphology, and identification of individual mosquito genera. The remaining pages are divided among dscussions of fleas, lice, bedbugs, triatomine bugs, cockroaches, mites, and argasid and ixodid ticks. Although such inequality in coverage seems prejudicial, it is partially justified by the relative epidemiologic importance of dipterans as vectors of arthropodborne diseases in humans.

This edition updates strategies for controlling insects, ticks, and mites. Extensive illustrations and color photographs may help readers recognize arthropods, such as mosquitoes, flies, and myiasis-producing larvae, and distinguish between soft and hard ticks. The book includes a glossary of entomologic and epidemiologic terms and a list of commonly used insecticides and their trade names.

Although the book is aimed primarily at students, it is also suitable as a general introduction into medical entomology for practicing physicians, nurses, public health officials, epidemiologists, and pest control professionals. The book also is valuable to anyone interested in learning basic information about the diversity and biology of medically important arthropods and ways of controlling them.

